# The impact of varying cursor latency on visuomotor tracking

**DOI:** 10.1007/s00221-026-07291-0

**Published:** 2026-05-02

**Authors:** Lucy J Turner, Steven D Wiederman, Jessica L O’Rielly, Anna Ma-Wyatt

**Affiliations:** 1 School of Biomedicine, Adelaide University, Adelaide, Australia; 2 School of Psychology, Adelaide University, Adelaide, Australia; 3 Andy Thomas Centre for Space Resources, Adelaide University, Adelaide, Australia

**Keywords:** Feedback, Online updating, Pursuit tracking, Latency

## Abstract

The visuomotor system compensates for sensory feedback delays, enabling the integration of visual information with motor commands to track moving objects with precision. However, when latency associated with a movement outcome becomes variable or noisy, this ability to compensate is tested. We aimed to identify the thresholds at which delays disrupt tracking to understand the limits of visuomotor prediction and error correction. Participants performed a visually guided pursuit task following a target along a curvilinear path, while eye and cursor movements were recorded under various latency conditions. Cursor position was recorded throughout each trial to quantify mouse-to-cursor latency, cursor velocity, and cursor–target error. In the first study, latency was constant within trials. Six latency magnitudes (0–300 ms) were tested and their impact on tracking performance analysed. Spectral analysis of cursor velocity showed that under high latency, pursuit submovements shifted to lower frequencies interspersed with high frequency corrective actions, whereas under low latency conditions, smoother tracking dominated. In the second study, abrupt changes in cursor latency were introduced mid-trial. Step increases in latency resulted in larger positional errors and increased corrective submovements, while decreases in latency led to rapid error reduction and a return to smoother tracking. These transitions highlight the visuomotor system’s ability to recalibrate in response to latency variability. Together these results show that error correction mechanisms rapidly respond to changes in latency depending on its magnitude. These results may inform the design of assistive display technologies that maintain performance under challenging conditions and increase safety in high-risk environments.

**Electronic supplementary material:**

The online version of this article (10.1007/s00221-026-07291-0) contains supplementary material, which is available to authorized users.

## Introduction

Neuronal signals in the visuomotor pathway are inherently noisy due to processing delays, errors in motor planning, and visual distractors. To manage these, the nervous system has evolved to compensate for a degree of delay and uncertainty. However, modern human-machine technologies can disrupt these compensation mechanisms. Information displays that aim to provide users with current information about their environment may introduce delays due to sensing, transmission, processing, and display time. Additionally, there may be noise in the arrival time which varies the delay magnitude at each sample. We know that a change in delay between action and visual feedback of the action consequence will impact reaction time and overall experience (Smith et al. 1960; Held et al. 1966; Vercher and Gauthier 1992; Arnold et al. 2012). However, in reality, latency amplitude is not perfectly constant and how a single change in latency impacts manual tracking behaviour is not well characterised in the literature. In this study, we begin to examine the human visuomotor response during manual tracking when an artificial delay has a single step change. A deeper understanding of how the brain adapts to changes in the delay timing can inform the design of display systems.

Previous literature has highlighted how errors guide motor adaptation. The literature differentiates between real-time errors, between the action consequence and the target, and sensory prediction errors, which represent the discrepancy between predicted and actual outcomes (van Beers et al. 1999). In this context, a sensory prediction error occurs when the brain’s expected sensory feedback, based on its internal model of a movement, differs from the visual or proprioceptive information that is actually received, signalling the need to update the motor command or sensory weighting. Sensory prediction errors are not directly observable, though they may be contained in an error signal when introducing perturbations or delays to a target. These manipulations amplify the natural discrepancies that the brain encounters in everyday life. By creating a mismatch between the predicted and actual position of the cursor on the screen, researchers can observe and investigate how the brain detects and corrects these errors. Research into error correction mechanisms, such as the work by Smeets et al. (2003, 2016) has shown how humans rely on both visual and proprioceptive feedback to correct for discrepancies during tracking tasks. Visual feedback provides rapid updates that guide movement corrections, while proprioceptive feedback offers information about limb position and movement, contributing to more refined motor adjustments (Brenner and Smeets 1997; Sober and Sabes 2003). Visuomotor tracking tasks provide a controlled way to study how the nervous system resolves conflicts between sensory modalities and updates internal predictions accordingly. Visual feedback can be delayed experimentally while proprioceptive feedback remains temporally accurate.

To reduce sensory prediction errors arising from delayed feedback, the motor system can employ predictive control strategies that estimate future states of the cursor. Vercher and Gauthier (1992) characterised how eye movements differ when following a memorised path with the cursor (invisible target) versus when following a visible target on the screen. In their study, a target moving sinusoidally along the horizontal plane elicited different eye movement strategies based on the memorised or visible condition. In the memorised path scenario, eye movements anticipated the screen update, relying on internal feedback such as efferent copies or proprioceptive signals rather than retinal slip. When a delay between the hand movement and the self-driven cursor was introduced, the eyes moved before any retinal slip could occur. In contrast, when the participants controlled a cursor tracking an unpredictable target, retinal slip provided the necessary feedback to adjust the eye movements to match the target’s unpredictable motion. Participants exhibited anticipatory movements to lead the target with the cursor, reducing the sensory prediction error that would otherwise arise; however, this anticipatory behaviour diminished when delays exceeded 300 ms. This research highlighted that these two conditions adopt distinct predictive systems, suggesting separate neural predictors operate depending on task demands. Together, these studies highlight that tracking behaviour emerges from the interaction between prediction and feedback correction, both of which are sensitive to feedback timing.

An interception task requires the participant to intercept a moving target, which differs from a reaching task where the target is static. Discrete movements in an interception task with cursor delays or jitter noise may provide insight into how a change in cursor latency during tracking will impact performance. During this task a single movement is made towards a moving target to intercept it. de la Malla et al. (2014) ran several interception tasks including manual tracking, passing through a moving gap, temporal judgement, auditory synchronisation, and a movement direction task. They found that participants learn to control delayed visual feedback within each task, however this adaptation did not transfer across to a new task. This task-specific learning indicates that individuals can fine-tune their movement to accommodate for a predictable delay and enhance their performance by adapting their movement over time. Interception tasks provide a contrast to our study paradigm of continuous tracking as they demonstrate predictive control under temporal constraints. This helps to clarify which aspects of delay depend on advanced planning and online correction as the window for online correction is limited.

In an interception task, Brenner and Smeets (2023) observed that jitter in the cursor is responded to similarly as jitter in the target; however, the responses are not as robust. This difference in motor correction strength is likely due to the proprioceptive feedback from the hand, which provides additional sensory information that helps mitigate the effects of visual jitter. The trials involved one planned movement towards an endpoint, which typically lasted around 500 ms. Participants appeared to adjust their movement based on how much time remained to reach the endpoint. This may reflect an update of their intended movement plan rather than relying solely on continuous online corrections. However, whether these movements constitute true replanning or simply fast corrections remains unclear. During manual tracking a combination of planned movements and online corrections are guided by an internal model to handle delays and uncertainty (Poulton and Edwards 1974; Wolpert et al. 1995; Miall et al. 1993).

A constant visual feedback delay during tracking will impact tracking performance, particularly before learning and adaptation occurs (Miall et al. 1985; Miall and Jackson 2006; Rohde et al. 2014). Interception requires a participant to predict and align their movement with a moving target with spatial and temporal precision. In a study looking at how gaze is impacted by delayed cursor feedback during an interception task, it was found that the gaze remained on the target (Cámara et al. 2018). The velocity signal is an important cue in tracking tasks as it helps the participant not only follow the target’s current motion but also anticipate its future path. It is therefore an essential cue for both real-time error and sensory prediction error. Furthermore, the ability to maintain gaze on the target while relying on velocity-based predictions supports a functional coordination between perceptual stability and the motor response. Gaze stability, predictive estimation and corrective motor responses interact to compensate for the imposed latency, providing insights into the compensatory mechanisms participants use to cope with feedback delays. However, these interception studies are largely based on discrete movements, leaving open the question of how similar mechanisms operate during continuous tracking and a sudden delay change.

Temporal recalibration refers to the brain’s ability to adjust its perception of time in response to external stimuli. If there is a change in feedback due to noise or delay, temporal recalibration can be seen in the aftereffects (Foulkes and Miall 2000). While we are not directly measuring this process, recalibration will contribute to the movement dynamics we observe. When a cursor delay is introduced, compensatory mechanisms initially fail. At around 100 ms delay, the frequency of corrective movements and their speed decreases (Foulkes and Miall 2000). This may lead to overshooting and more rapid changes in position, measured as increased amplitude at higher frequencies in the velocity data (Foulkes and Miall 2000). A reduction in movement amplitude relative to the target indicates undershooting (Rohde et al. 2014). The term intermittency, used to describe these stop-start movements, comes from intermittent control systems that employ an on/off mechanism, whereby the device is turned off until an error threshold is reached and corrected for. When humans track a target with a cursor, this appears as peaks in the velocity profile of the movements (Craik 1947; Susilaradeya et al. 2019). During this intermittent “move and wait” strategy, visual inputs are ignored while the motor command is executed. Movement is then paused while feedback arrives, and an error correcting movement is made. If errors are calculated based on the hand movements when there is a visual feedback delay, an error equivalent to the delay condition is required to track or acquire a target successfully (Cámara et al. 2018). When a delay is increased from 59 to 200 ms by 1ms over each trial, participants adapted to the delay by moving their finger ahead of their gaze (Cámara et al. 2018). While adaptation may transfer across tasks, for example reaching to tracking, compensation may not generalise over to different tasks, instead the enactor will learn the delay compensation as a consequence of a particular sensorimotor task (de la Malla et al. 2014).

Motor submovements, components of a smooth action, can be characterised by looking at variability in the movement velocity from a tracking task. When tracking a slow circular target, the primary peak in the power spectra of the velocity occurs at approximately 2 Hz due to an intrinsic delay of the motor system (Susilaradeya et al. 2019). When an additional artificial delay of 200 ms is added to the cursor, the primary peak shifts to 1.4 Hz and harmonics are introduced. Peaks in the power spectra may be explained by constructive interference between intrinsic motor system properties and the extrinsic delay of feedback and associated compensatory corrections. When participants track a moving target, they generate motor commands based on the visual feedback they receive. However, the natural delay in the feedback loop due to processing time in the sensory and motor systems means that the corrective movements are based on slightly outdated information about the cursor’s position relative to the target. If a corrective movement (intended to reduce an error) occurs when the target has already changed direction, it can add to the new error, resulting in larger, more pronounced submovements. So when the feedback delay is higher, corrective submovements occur less frequently and the temporal structure of corrections changes, reflecting an intermittent control strategy rather than continuous online correction (Susilaradeya et al. 2019). However, the question remains of how the temporal structure of these submovements is reorganised when feedback timing is abruptly altered.

External sources of delay introduced by technology, such as display systems, sensors, and network infrastructure, play a critical role in shaping feedback timing and user performance. If a delay is constant, a participant can adapt to the delay up to a certain magnitude. However, both local device or system latency (e.g., processing or rendering delays) and network latency (e.g., internet transmission delays) can contribute to variable delay (McCabe 2007; Wu et al. 2013; Høiland-Jørgensen et al. 2016). Asynchronous system components, timestamp limitations, and buffering inconsistencies may contribute to variability in the feedback timing. Display latency associated with a head-mounted or near-eye display can be time-varying. For instance, noise in the inertial measurement unit (IMU), such as random fluctuations in gyroscope or accelerometer readings, can introduce errors in estimating head orientation, leading to unstable or delayed visual updates. More recently, the effect of time-varying delay on visuomotor behaviour has been investigated (St. Pierre et al. 2015; Kinsella et al. 2016), though much of this work focuses on simulator sickness rather than performance. Many of these tasks use short, discrete movements rather than continuous control that depends on real-time feedback. Kinsella and Hoover (2016) found that the frequency of delay variation (0.2 Hz), rather than its amplitude (20–100 ms), had a stronger influence on reported sickness. Wilson et al. (2020) used a visual search and target shooting task, finding that performance (hit time and accuracy) was better under constant than varying delay (70–270 ms at 0.2 Hz). Beadle et al. (2021) reported that performance improvements could occur even in the presence of sickness, with participants adopting a “move and wait” strategy to compensate. Together, these findings highlight a gap in our understanding of how time-varying feedback delays influence the frequency and amplitude of corrective submovements during continuous control.

What is not well understood in the literature is the frequency and amplitude of submovements in response to a feedback timing change. This study investigates what movement occurs during and following a single cursor latency change (increase or decrease). The lowest latency condition begins at 100 ms, the approximate time that it takes to make a correction with the hand using visual information (Foxe and Simpson 2002; Saunders and Knill 2005). Delays larger than 400 ms become too detrimental to performance (Foulkes and Miall 2000) so the highest latency is limited to 300 ms where performance drops substantially (Miall and Jackson 2006). A 2D display was used to investigate the effects of latency on visuomotor performance. Many visuomotor experiments use linear trajectories, however, curvilinear trajectories simulate motion in the natural environment. It has been demonstrated that participants are able to implicitly learn repeated curvilinear segments during a pursuit tracking task using a joystick (Pew 1974; Ewolds et al. 2017; Broeker et al. 2020). A movement pattern may be learned within a single session of practice (Ewolds et al. 2017). To minimise early adaptation or tracking errors driven by curvilinear motion, participants completed 20 practice trials prior to the main experiment.

We use a continuous pursuit tracking task while recording eye and hand tracking. The performance measures include Euclidian distance between target and cursor, the error corrected for target direction, and the cursor velocity profile. The visuomotor system adapts when environmental cues are limited or altered, rather than continuously optimising which is not energy efficient (Raab et al. 2013). We are interested in whether a similar approach will be adopted by participants in our study in response to a single step change in latency. This is the first step to identifying how time-varying feedback may continue to impact movement control. The objective was to understand how the step change in latency altered the participants’ movement patterns. It outlines the short-term motor adaptation that takes place following a latency change including sudden changes in submovement strategy.

## Methods

### Apparatus

**Fig. 1:**
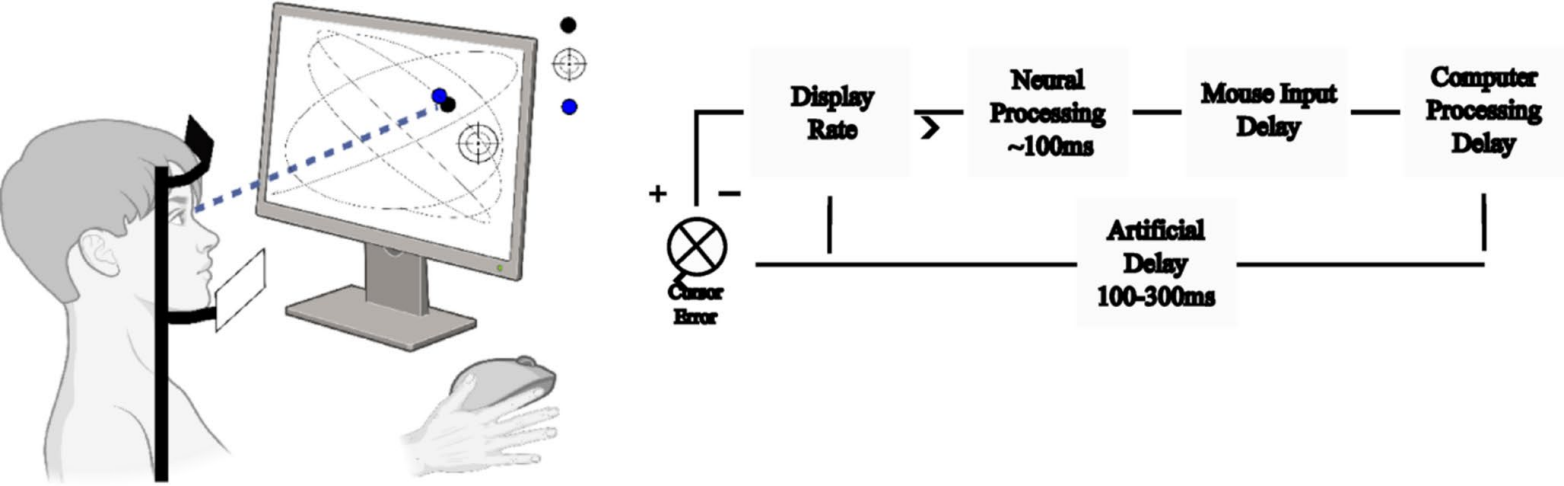
Experimental set up and latency pipeline. Left panel: The gaze (blue dot, not shown to participant) tracks the target with the goal of maintaining the cross hairs over the target. The target has equidistant waypoints and moves at approximately 8°/second. Right panel: box diagram of motor input, mouse polling, and cursor movement. (adapted from Poulton 1974). This demonstrates the point at which the visual processing delay and artificial cursor latency occurs.

A 17′′ Elo monitor 1729 L (1024 × 1280) at 75 Hz was used to present the stimuli and a Logitech gaming mouse was used with the input device set at 1000 DPI. Eye movement data was collected at 1000 Hz using the SR Research Eyelink 1000 (Fig. 1). Participants rested their chin on a chinrest at 57 cm distance from the screen and their eye position was calibrated at the beginning of each block of trials (20 trials per block) using a 9-point calibration.

### Visual stimuli

**Fig. 2:**
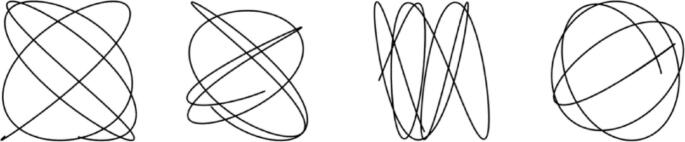
Four curvilinear paths were rotated and reversed for a total of 12 paths. Waypoints processed to be equidistant.

The visual stimulus consisted of a black dot target (1.9° visual angle) moving along a curvilinear path across the screen. The cursor appeared as a crosshair. The white background had a luminance of 173 cd/m^2^. Four target paths were made from a sum of three sinusoids for the X coordinates and a sum of three different sinusoids for the Y coordinates (Fig. 2). Four additional paths were generated by rotating the original four by 90°. A further four paths were generated by reversing the order of the coordinates. This method ensured that the participant experienced similar features throughout the 12 different paths. Changes in direction occurred near the edge of the screen as a gradual curve. The screen edges can be used as a reference point for the participant who can then anticipate a direction change (Kowler et al. 1984). The paths were normalised to the screen and the waypoints were equidistant. Participants were required to click the mouse to begin each 30 s trial enabling them to have a short rest when they needed, whilst keeping their chin on the chin rest. After 20 trials (10–15 min) participants took a 5-minute break before recalibrating for the next block. The participant controlled a cross-hair using the mouse. The positions of the target and the crosshair were sampled at 75 Hz. The experiment was written in MATLAB using Psychophysics Toolbox (Brainard 1997).

### Pursuit tracking task

The participant viewed the target moving along a curvilinear path and was required to manually align the crosshair over the target using the mouse. The time that it took for the crosshair position to update following the mouse movement was manipulated across conditions with an artificial delay (Fig. 1). All trials were 30 s long.

In the first experiment, participants had a practice block during which they were exposed to the control condition and the highest latency condition (300 ms). There were 5 repeats of each condition with a total of 10 trials within the practice block. This was to ensure the participant understood the task demands and to observe that they were capable of performing the highest latency condition in the task. Capability meant that they were able to complete the trials without asking to stop and without disengagement or prolonged cursor inactivity. No participants were excluded at this stage. In the first experiment, the crosshair latency remained consistent, and five latencies were tested (100 ms, 150 ms, 200 ms, 250 ms, and 300 ms) as well as a control condition. The rationale for selecting these specific latencies is outlined in the introduction. To counterbalance latency order across participants, the sequence of latency conditions was reversed for alternating participants. For the 6 conditions there were 10 repeats of each condition with a total of 60 trials.

In the second experiment, the crosshair latency increased or decreased by one of four magnitudes: 150 ms, 200 ms, 250 ms, or 300 ms. This occurred halfway through the trial at 15 s. For the 8 conditions there were 10 repeats of each conditions with a total of 80 trials. The participant was aware that the crosshair dynamics might change, although they were naïve to the amount of latency that would be present.

### Data analysis

Data analysis was conducted in MATLAB 2022a with support from the Statistics and Machine Learning Toolbox. Statistical data visualisation was generated using the Seaborn, SciPy, NumPy, and Matplotlib packages in Python and edited using Inkscape 1.2.2.

### Smooth pursuit error

**Fig. 3:**
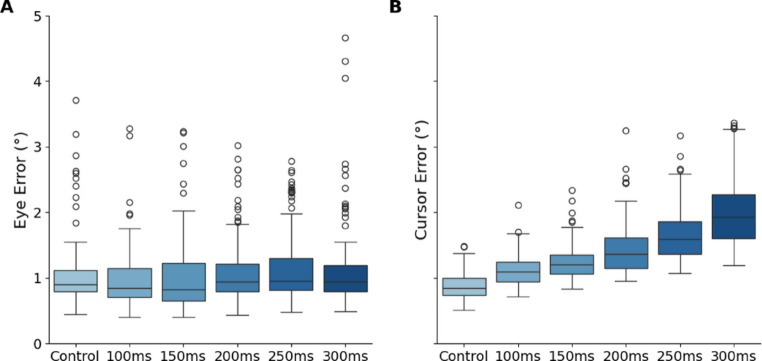
Box and whisker plot of all participants (*n* = 14) from the first experiment with median and interquartile range (25th − 75th percentiles). Whiskers extend to 1.5 × IQR. **A** The average eye/target error magnitude (smooth pursuit accuracy) does not change with cursor latency. **B** The trial average cursor/target error magnitude increases with cursor latency.

Trials with more than 200 ms of data missing were excluded due to data collection artefacts (loss of pupil due to glasses, fidgeting, or other). This removed 34/840 trials across participants (4%). Smoothing was performed using a symmetric moving-average window centred on each sample (± 3 ms). The first second of initiation was excluded from each trial to ensure an equal length of samples. The accuracy of smooth pursuit was measured as the Euclidean distance between eye and target position which was averaged across each trial (Fig. 3A). A linear mixed model was used to examine the relationship between eye/target error (accuracy) and latency, accounting for individual variability. Fixed effects in the model included latency, while participant variability was modelled as a random effect as variance components. The model specification was: *eye error ~ latency + (1 | participant)*.

### Cursor error

The raw 2D position data of the cursor on the screen was processed as follows. The first second of cursor pursuit initiation was excluded. The Euclidean distance between the target and the center of the crosshair was calculated for each sample. In the first experiment, this was averaged over the trial. In the second experiment, to investigate the overall trend in error during the event-related change in latency, for each sample the mean error was computed across repeats (time-synchronous average). An across-participant mean, and SEM was then calculated (Fig. 7).

To find out if there was a relationship between latency and cursor error, error magnitude was used in a statistical analysis. A linear mixed model was used to examine the relationship between cursor/target error and latency, accounting for individual variability. Fixed effects in the model included latency, while participant variability was modelled as a random effect as variance components. The model specification was: *cursor error ~ latency + (1 | participant)*. Next, to find out if there was a bias in the direction of error (i.e. lagging or leading), the directional velocity was used to rotate the error vector and find cursor error expressed in a target-centred coordinate system, with analysis focused on the y-axis component. This will be referred to as “y-axis error”. Fixed effects in this model included latency, while participant variability was included as a random effect. The model specification was: *y-axis error ~ latency + (1 | participant)*.

The velocity and cursor-target error relationship was examined across the six latency conditions. Because error data were positively skewed, a Spearman correlation was used and a robust linear model (M-estimator with bisquare weighting) was employed to mitigate the influence of outliers.

### Velocity and spectral analysis

A second order 10 Hz Butterworth filter was applied to the speed trace, and the data was smoothed with a Hanning window to reduce effects of spectral leakage. There were 2175 samples per trial which were padded with zeros up to the next power of two (4096). A fast Fourier transform (FFT) was then used to compute the amplitude spectrum, capturing the frequency content of movement fluctuations or submovements. This was computed for each trial, averaged across repeats, and then across participants. Peaks in the spectra were identified using the *findpeaks* function in MATLAB.

### Participants

Participants were recruited from the Adelaide University School of Psychology and were reimbursed with course credit. The experiment included 11 females and 3 males (18–43 years) who participated in two one-hour sessions (described under *Pursuit Tracking Task*). One participant was left-handed; all other participants were right-handed. All participants had normal or corrected to normal vision, as well as no history of neurological disease or a diagnosed motor impairment. As task performance relies on the visual input, participants were screened using the following standard testing protocols in vision science. The Pelli-Robson chart was used to screen for deficits in contrast sensitivity and the Snellen chart was used to test visual acuity. Ethics were approved by the School of Psychology Human Research Ethics Subcommittee. All participants gave informed consent and were free to withdraw at any time without penalty.

## Results

### Cursor error in experiment one

**Fig. 4:**
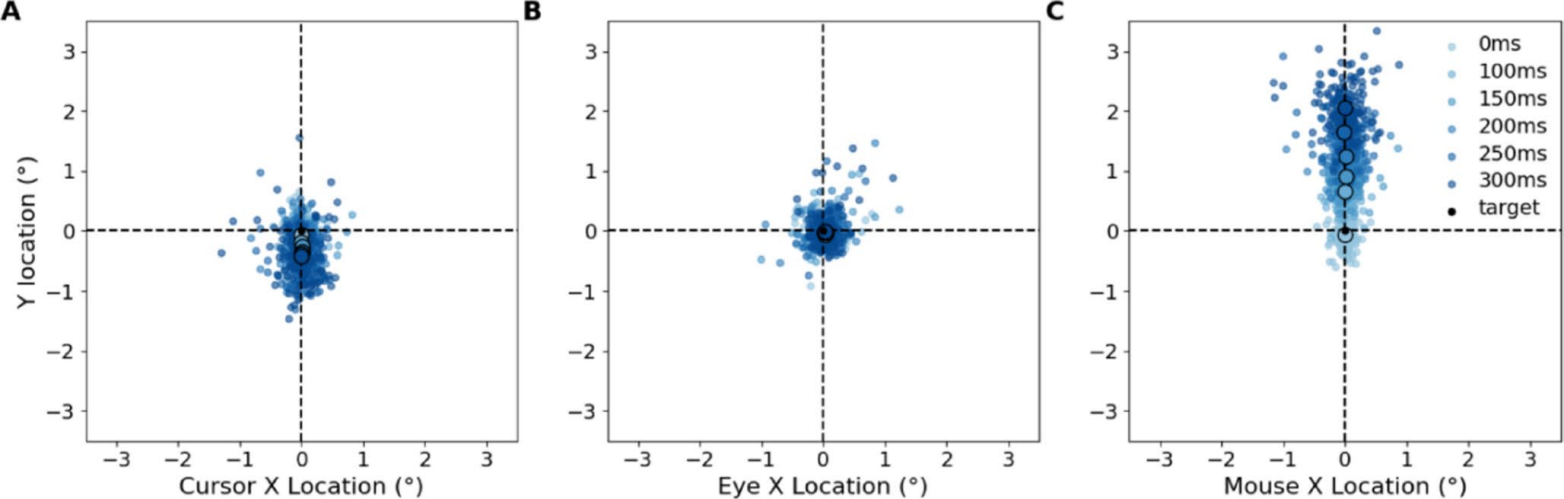
All participant data from the first experiment. The origin represents the target location. Each dot represents the average error per trial after being corrected for direction. **A** The error between the cursor feedback on the screen and the target has a negative (lagging) bias. **B** The average directional error per trial between the eye and the target does not have a bias in direction across conditions. **C** The participant compensates for the artificial latency by moving the mouse and making predictions ahead of the target.

To evaluate how increasing latency impacts manual tracking performance, we assessed cursor-target error across varying delay conditions. Participants maintained close visual alignment with the target across all conditions, with average gaze remaining within 2° of the target, and 99.1% of trials within 1° (Fig. 4B). This confirmed that participants consistently monitored the target throughout the trial. A linear mixed-effects model (LMM) was used to test the effect of latency on cursor-target error magnitude. As the cursor latency increased the average cursor error magnitude increased (Fig. 3B). A Type III ANOVA (Satterthwaite’s method) on the linear mixed-effects model indicated that latency had a significant effect on cursor error across all conditions (*p* < 0.001). Individual differences contributed meaningfully to the overall model fit. The marginal *R*2 value R^2^_m_ represents the proportion of variance in the cursor error that is explained by the fixed effects alone. In this model, R^2^_m_ is 0.53, indicating that 53% of the variability can be attributed to the latency. The conditional R2 accounts for both the fixed and random effects, reflecting the total variance explained by the model. Here, R^2^_c_ is 0.83, suggesting that the combination of latency and individual differences explains 83% of the variance in the cursor error. A model comparison revealed that adding a second random effect of target path did not improve the model AIC_part_path = 0.37098 compared to AIC_part = 1.7394.

Next, to find out if there was a bias in the y axis error or if the cursor error was equal in lagging and leading, we fit the y-axis component of the average cursor error to a mixed model. The analysis revealed a significant effect of latency on the y-axis variable (*F*(5,816.95) = 44.37, *p* < 0.001). However, there was no significant intercept effect (F(1,18.27) = 1.04, *p* = 0.32). The values for the pseudo *R2* measures indicate that the latency alone explains approximately 13.5% of the variance in the dependent variable R^2^_m_ *=* 0.1353, while the model accounts for 49% of the variance R^2^_c_ = 0.49). These results suggest that while the latency contributes to explaining the variance in the y-axis error, a substantial amount of the variance is captured when including individual differences.

The fixed effects coefficients indicate significant differences across conditions. 100 ms latency showed a significant reduction in the response variable relative to the reference condition (*β* = − 0.13, *t*(834) = − 4.60, *p* < 0.001). 150 ms latency showed a further reduction (*β =* − 0.20, *t*(834) *= −* 6.95, *p <* 0.001). Latency 200 ms, 250 ms, and 300 ms each showed progressively larger reductions in the response variable, with 300 ms showing the largest effect (*β* = − 0.35, *t*(834) *= −* 12.51, *p <* 0.001). These results indicate a systematic increase in the average y axis error value as latency increases (Fig. 4A), which reflects an increase in lag on the screen between the cursor and the target.

### Velocity and spectral analysis

**Fig. 5:**
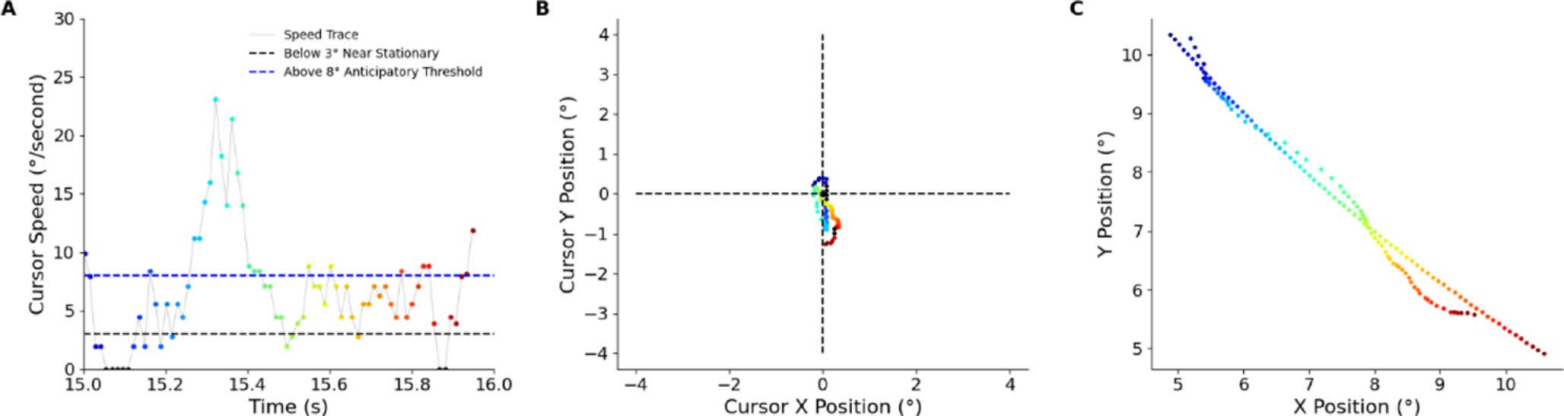
Example of one participant in the control condition. Colour represents time within the epoch in panel A and is preserved across panels B and C to indicate corresponding temporal samples. Black dots represent cursor velocity below 3°/s. **A** The cursor speed over time shows that movements are exaggerated when the initial correction is wrong. **B** The cursor maintains the target with some accuracy. **C** The error amplitude remains steady.

Submovements appear as fluctuations in the velocity data and a different movement pattern is observed in each of the constant latency conditions in the first experiment. To examine whether participants engaged in predictive control, we analysed cursor movements relative to target motion. When corrected for direction the cursor typically deviates from the origin with a lag and side to side error to maintain proximity to the target (Fig. 4A). When the cursor begins to lag a quick movement occurs to correct the position (aqua/light blue data points Fig. 5).

**Fig. 6:**
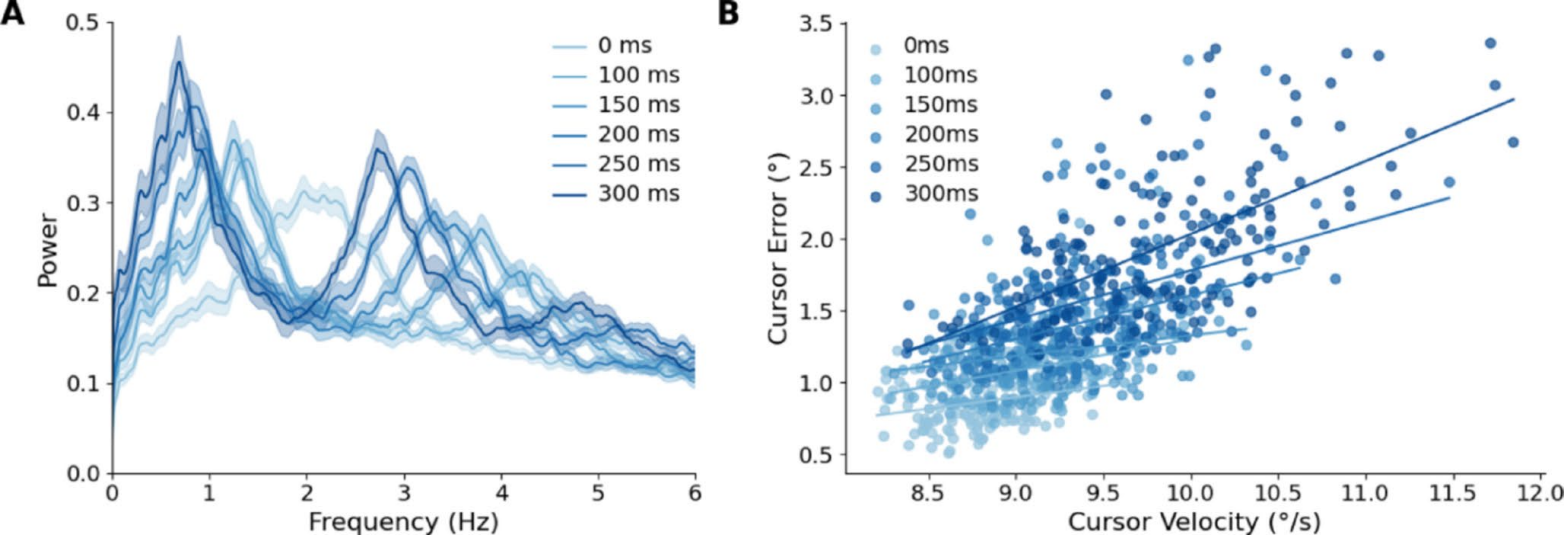
Frequency spectrum of cursor velocity. **A** The amplitude at each frequency (derived from the magnitude of the FFT) was averaged across participants and SEM is shaded. As the latency increases, the fundamental frequency shifts to the left. A third harmonic appears in the 100 ms latency condition. A fifth harmonic appears in the 250 ms condition. **B** Cursor error versus cursor velocity for each latency condition. Each point represents a single trial, and the solid lines indicate robust linear fits within each condition. Error generally increases with velocity, and the slope of this relationship becomes steeper as latency increases, indicating that feedback delays exacerbate velocity-related overshoot.

The fundamental frequency of the velocity trace occurs at 2.15 Hz ± 0.29 in the control condition, whereas it occurs at 0.61 Hz ± 0.13 in the 300 ms condition (Fig. 6). This demonstrates a lower frequency of submovements in high latency conditions. A one-way ANOVA of the fundamental frequency in the velocity with Bonferroni corrections revealed significant differences between the control and all latency conditions (F(5,78) = 180.05, *p* < 0.001). However, there was no significant difference between the 150 and 100 ms conditions (*p* = 0.062) or between the 150 ms and 200 ms conditions (*p* = 0.788). 250 ms latency was not significantly different from the 300 ms condition (*p* = 0.646).

The higher velocity-error correlations at longer latencies suggest the relationship is getting stronger with increased delay. Spearman’s ρ moves from 0.24 at 0 ms latency to 0.66 at 300 ms latency. The slope tends to increase as latency increases from 0.16 at 0 ms to 0.51 at 300 ms and the intercept becomes more negative as latency increases from 0.53 at 0 ms to 3.04 at 300 ms.

### Experiment two

**Fig. 7:**
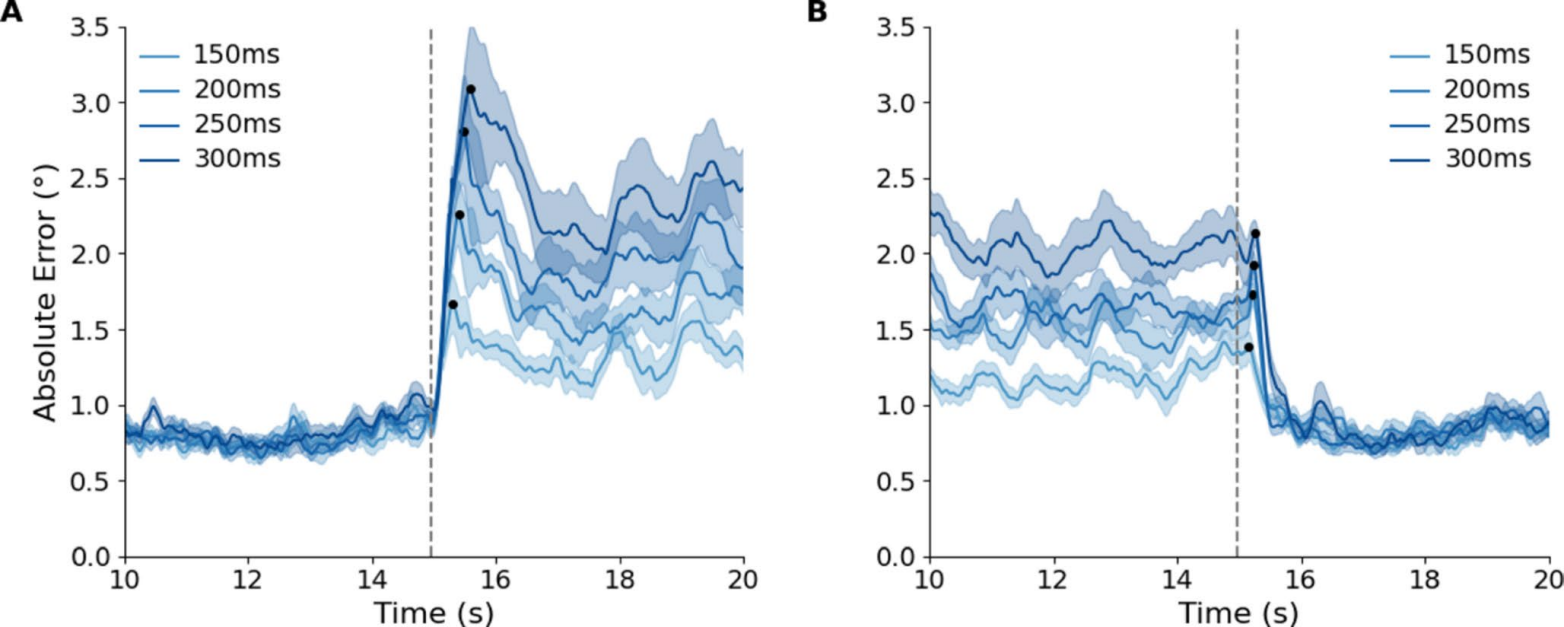
The time-synchronous average in cursor error magnitude across all participants with the SEM shaded. The latency change occurs at the dashed line at 15 s. **A** The average error when there is a step increase in latency. **B** The average error when there is a step decrease in latency.

To examine how quickly participants adapt their tracking behaviour following a step change in cursor latency, we analysed the time course of error immediately after the latency shift. In the cursor latency increase condition, there is an overall increase in error following the change in cursor latency, which stabilises within 1 s or closer to 1.5 s at the higher latency. In the cursor latency decrease condition, there is a noticeable drop in error across all latency magnitudes. The drop in error occurs within 0.5 s across all conditions. By averaging across participants and repeats at the same time point, the fluctuations in error are reduced. This describes the average error amplitude made following the latency change (Fig. 7).

**Fig. 8:**
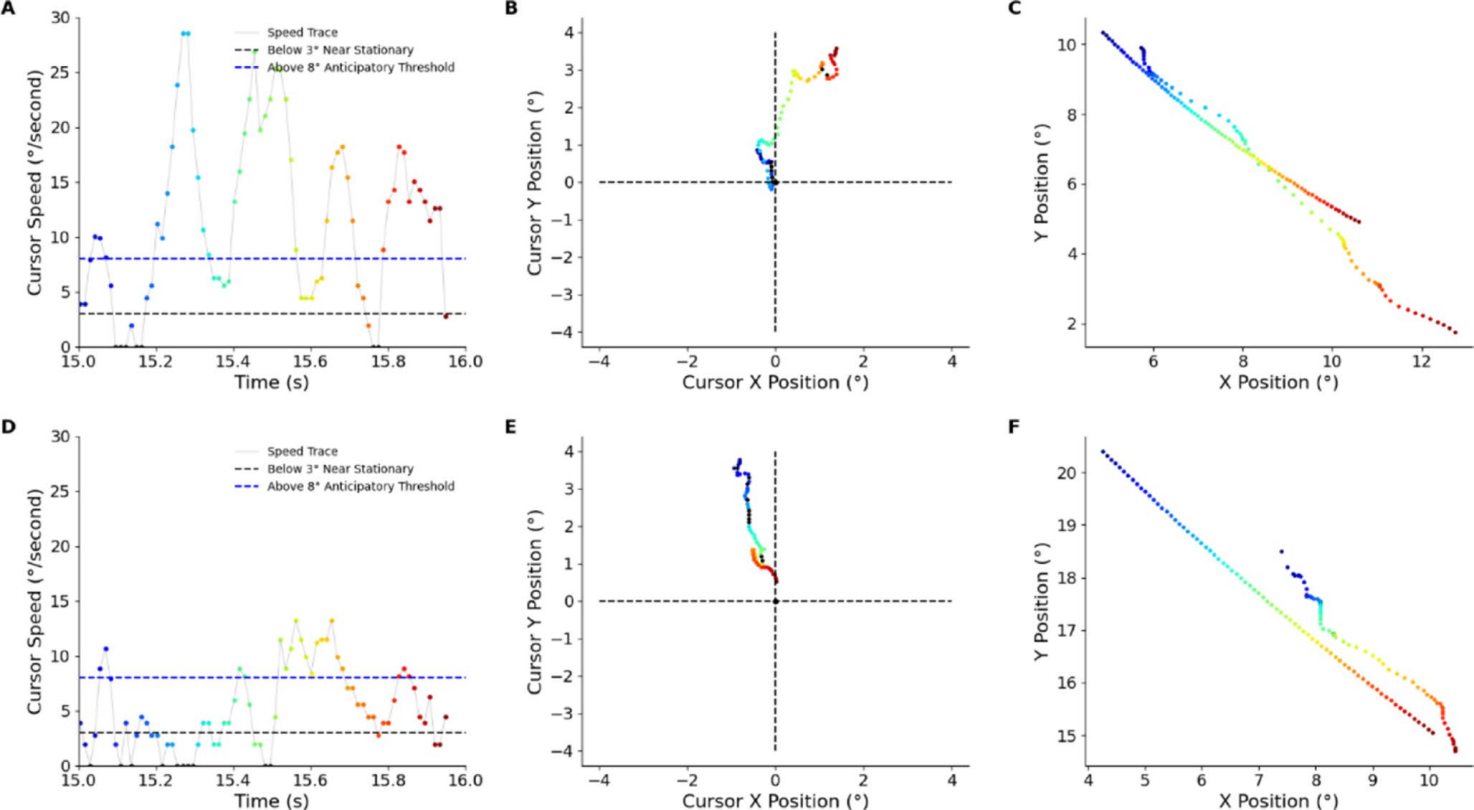
Example from 1 s of a 30 s trial from a single participant following an increase in latency by 300 ms (top row) and a decrease in latency by 300 ms (bottom row). Colour represents time within the epoch in panel A and is preserved across panels B and C to indicate corresponding temporal samples. Black dots represent cursor velocity below 3°. **A** The increase leads to a spike in cursor speed with smaller fluctuations (corrections). **B** The increase in latency leads to an increase in y-axis error ahead of the target or overshoot. **C** An increase in latency leads to increased error amplitude. **D** A rapid adaption occurs in response to decreasing latency with smaller spikes in velocity. **E** The decrease in cursor latency leads to a decrease in y-axis error. **F** A decrease in latency leads to reduced error amplitude.

## Discussion

In this study, we investigated how a step change in latency impacts participants’ movement patterns during a manual tracking task. Using the measures of error magnitude, directional error, and velocity we described the short-term behavioural response to an abrupt step change in cursor latency. Even in the control condition, where the latency was minimal for this system, tracking was not perfectly smooth. When introducing a larger artificial latency, participants make larger, low frequency movements along with small corrective movements in efforts to compensate for the delay which results in greater errors. These findings align with earlier research (Craik 1947; Miall 1996; Susilaradeya et al. 2019), reinforcing the idea that participants adapt their movement strategies to cope with delayed feedback, at the cost of accuracy in the early stages of adaptation.

A key observation in the velocity trace was the intermittent time spent near stationary (the black data points in Figs. 5 and 8) which raised the question: are participants waiting on a slow target or were they adopting the “move and wait” strategy seen in intermittent control systems? Susilaradeya et al. (2019) investigated two different target speeds over a circular trajectory: slow (0.1 cycles/s) or fast (0.2 cycles/s). The frequency of the cursor movements increased in the control condition when the target moved faster. However, in the 200 ms cursor latency condition there was no significant change. The same movement strategy was adopted to handle the artificial latency for both target speeds. This suggests that the observed intermittency is more of a delay-dependent control strategy rather than a response to target dynamics.

During pursuit tracking, participants will focus on the target, using their peripheral vision to monitor the cursor (Cámara et al. 2018; Mathew et al. 2019; Brenner and Smeets 2023). This suggests that velocity information is critical for smooth pursuit. However, when velocity information becomes unreliable as an anticipatory cue, a position cue can take precedence for real-time correction (Miall and Jackson 2006; Parker et al. 2021). In our study, velocity information becomes unreliable as it is temporally misaligned with the current state due to feedback delay. As cursor latency increases, participants shift to a more intermittent control strategy, characterised by larger, less frequent corrective movements, especially when tracking unpredictable targets. Parker et al. (2021) fitted two models with behavioural data consisting of two conditions: pseudorandom/unpredictable target tracking and sine wave/predictable tracking. The modelling demonstrated that when a target follows predictable motion, participants can anticipate its movement using negative feedback control biased by velocity. In contrast, an unpredictable target requires adaptive control that operates intermittently.

Our findings can be interpreted within existing models of delayed sensorimotor control. Visuomotor control already contends with an inherent visual processing delay of approximately 100–150 ms (Carl and Gellman 1987). Early models, such as the Smith predictor, suggested that the cerebellum could compensate for this delay by predicting the outcome of movements (Miall et al. 1993). However, Miall and Jackson (2006) questioned this model, and showed that while participants adapt to delayed feedback over time, their movement intermittency does not return to pre-delay levels. Instead, the larger corrective movements become more accurate, suggesting that feedforward processes improve with practice, rather than switching back to velocity biased processes. Additionally, the ability to adjust to spatial perturbations can also be explained by internal models that update predictions based on noisy or delayed feedback. The Kalman filter is one such model, weighting the uncertainty of incoming sensory information to refine the internal estimate of the current state (Burge et al. 2008). This process allows participants to adapt to delays by adjusting their predictions rather than solely relying on real-time feedback. The intermittent movements observed in our study along with the ability to switch to new dynamics following a step change in delay is consistent with inverse dynamics where motor commands are mapped from desired motion (Takamuku and Gomi 2015).

Several task-specific factors may have influenced the observed behaviour. Although a mouse was used as the input device in this experiment, future studies could benefit from other input modalities, such as joysticks or isometric finger force devices. Such devices reduce artefacts from rigidity of the wrist and directional preferences of the mouse. However, the computer mouse is commonly used among our study cohort, allowing task learning time to be quite short. Another consideration is that the cursor (crosshair) size was larger than the target. This may have influenced participants’ perception of error, as they might have been satisfied with the edge of the cursor touching the target, rather than aiming for the centre. We chose to use crosshairs as they offer a reference frame around the target, which may encourage more accurate predictions of its movement compared to a simple dot, which lacks these spatial cues. This aligns with findings showing that visual structure improves tracking by facilitating motion extrapolation and proactive responses (Rosenberg et al. 1989). However, the resulting differences in strategy may explain some of the inter-individual variability in performance as described in the mixed models.

Finally, the asymmetry of increasing and decreasing delays warrants further investigation. While participants adjusted to increasing delays by adopting a more intermittent control strategy, the effects of decreasing delay may reveal additional insight into how the visuomotor system recalibrates to a more favourable condition. Overall, the study findings support the notion that the visuomotor system relies on both feedforward and feedback mechanisms to adapt to delayed visual feedback. Intermittent control strategies driven by position errors were used as the latency increased. Further research should continue to explore the neural and behavioural mechanisms underlying these adaptations with the goal of improving the design of human-machine interfaces and enhancing our understanding of visuomotor control in dynamic environments.

## Electronic supplementary material

Below is the link to the electronic supplementary material.

Supplementary file 1

## Data Availability

The datasets generated and analysed during the current study are available from the corresponding author on reasonable request, subject to approval by the University of Adelaide Human Research Ethics Committee (approval number 23/08).
