# Supplementary material for: The impact of varying cursor latency on visuomotor tracking
Source: Exp Brain Res. 2026 May 2;244(6):105. doi: 10.1007/s00221-026-07291-0 (PMC13135551; doi:10.1007/s00221-026-07291-0)
Supplement: Supplementary file 1 [file 221_2026_7291_MOESM1_ESM.docx]

Supplementary details

*Visual Stimuli*

$$xposes=xposes+\sin\left( 2\pi F_{i} \right)\times t- P_{i}$$

$$yposes=yposes+\sin\left( 2\pi F_{i} \right)\times t- P_{i}$$

| **Path** | **Frequencies X** | **Frequencies Y** | **Phase X** | **Phase Y** |
| --- | --- | --- | --- | --- |
| 1 | 0.01, 0.99, 0.99 | 0.81, 0.81, 0.81 | 9.72, 8.60, 8.60 | 4.39, 3.59, 3.59 |
| 2 | 0.99, 0.99, 0.014 | 0.81, 0.81, 0.81 | 1.00, 0.00, 0.00 | 1.00, 0.00, 0.00 |
| 3 | 0.19, 0.65, 0.05 | 0.99, 0.99, 0.98 | 1.00, 0.00, 0.00 | 1.00, 0.00, 0.00 |
| 4 | 0.81 ,0.90, 0.61 | 0.12, 0.91, 0.02 | 25.26, 0.00, 5.87 | 0.00,  2.46, 6.78 |

*Table 1: Frequencies and phases used for all four target paths. The way points were processed to be equidistant. The paths were then rotated or reversed to make up 12 total paths.*
